# Transcriptome alteration in *Phytophthora infestans* in response to phenazine-1-carboxylic acid production by *Pseudomonas fluorescens* strain LBUM223

**DOI:** 10.1186/s12864-018-4852-1

**Published:** 2018-06-19

**Authors:** Roxane Roquigny, Amy Novinscak, Tanya Arseneault, David L. Joly, Martin Filion

**Affiliations:** 1 Department of Biology, Université de Moncton, Moncton, Canada; 2 Saint-Jean-sur-Richelieu Research and Development Center, Agriculture and Agri-Food Canada, Saint-Jean-sur-Richelieu, Canada

**Keywords:** *Phytophthora infestans*, *Pseudomonas fluorescens*, Phenazine-1-carboxylic acid, Transcriptomics, Biocontrol

## Abstract

**Background:**

*Phytophthora infestans* is responsible for late blight, one of the most important potato diseases. Phenazine-1-carboxylic acid (PCA)-producing *Pseudomonas fluorescens* strain LBUM223 isolated in our laboratory shows biocontrol potential against various plant pathogens. To characterize the effect of LBUM223 on the transcriptome of *P. infestans*, we conducted an in vitro time-course study. Confrontational assay was performed using *P. infestans* inoculated alone (control) or with LBUM223, its *phzC-* isogenic mutant (not producing PCA), or exogenically applied PCA. Destructive sampling was performed at 6, 9 and 12 days and the transcriptome of *P. infestans* was analysed using RNA-Seq. The expression of a subset of differentially expressed genes was validated by RT-qPCR.

**Results:**

Both LBUM223 and exogenically applied PCA significantly repressed *P. infestans’* growth at all times. Compared to the control treatment, transcriptomic analyses showed that the percentages of all *P. infestans*’ genes significantly altered by LBUM223 and exogenically applied PCA increased as time progressed, from 50 to 61% and from to 32 to 46%, respectively. When applying an absolute cut-off value of 3 fold change or more for all three harvesting times, 207 genes were found significantly differentially expressed by PCA, either produced by LBUM223 or exogenically applied. Gene ontology analysis revealed that both treatments altered the expression of key functional genes involved in major functions like phosphorylation mechanisms, transmembrane transport and oxidoreduction activities. Interestingly, even though no host plant tissue was present in the in vitro system, PCA also led to the overexpression of several genes encoding effectors. The mutant only slightly repressed *P. infestans’* growth and barely altered its transcriptome.

**Conclusions:**

Our study suggests that PCA is involved in *P. infestans’* growth repression and led to important transcriptomic changes by both up- and down-regulating gene expression in *P. infestans* over time. Different metabolic functions were altered and many effectors were found to be upregulated, suggesting their implication in biocontrol.

**Electronic supplementary material:**

The online version of this article (10.1186/s12864-018-4852-1) contains supplementary material, which is available to authorized users.

## Background

The oomycete *Phytophthora infestans* is the causal agent of late blight of potato. With 240 megabases and more than 17,700 genes [1], the *P. infestans* genome is the largest of the *Phytophthora* genus. It contains highly repetitive DNA regions rich in mobile transposable elements and enriched with genes encoding proteins involved in pathogenicity and virulence [2]. Among these are effectors, including RXLR (~ 563 genes) and Crinkler (CRN) (~ 196 CRN genes), that are defined as molecules that can alter plant physiology and suppress immunity [3], therefore contributing to infection and disease development [4–6]. Transcriptional studies have shown that RXLR genes [7], as well as some CRN genes [8] are generally upregulated during the early stages of plant infection, while other CRN genes are upregulated at later stages during disease progression [8]. The association of effectors with transposable elements confers a highly adaptive flexibility to *P. infestans* [1], which accelerates the evolution of virulent isolates that render disease resistance genes in host plants ineffective. Moreover, the apparition of isolates resistant to commonly used fungicides, such as mefenoxam, makes control of the disease difficult to achieve [9, 10].

Despite the numerous studies that have been conducted on host resistance to *P. infestans*, only limited progress has been achieved in durably controlling the disease, in part due to its fast evolution and adaptive capacity [11]. Recently, studies were performed on *P. infestans* transcriptomic changes occurring during its life stages [12] and compared to *Pythium ultimum* during potato tuber colonization to identify potential targets for chemical control of the disease [13]. The results revealed that the transcriptome of *P. infestans* was very dynamic between growth stages, especially during spore formation and germination, where genes involved in pathogenicity, signaling and metabolism were differentially expressed [12]. The activity associated with some metabolic pathways was higher in mycelia than in spores, such as the pentose phosphate pathway, the tricarboxylic acid cycle (TCA), and amino acid and purine metabolisms [12]. Nutrient acquisition through mycelia structures, involving the glycolytic and gluconeogenic pathways, as well as pyrimidine uptake in *P. infestans*, has been shown important for plant pathogen’s growth, infection and disease establishment [14–16].

However, with the increasing public awareness and reluctance to use chemical pesticides, more environmentally friendly approaches are gaining in popularity. One of these approaches is biocontrol, which relies on the use of beneficial microorganisms for controlling disease development. Among biocontrol agents of interest, *Pseudomonas* spp. are known for their production of antibiotics involved in biocontrol, such as 2, 4-diacetylphluoroglucinol and phenazines [17–19], which have been widely studied in various plant-pathogen systems. Phenazine-1-carboxylic acid (PCA)-producing *Pseudomonas* spp. have been found effective against numerous plant pathogenic organisms, including bacteria, fungi, and oomycetes, such as the causal agent of bacterial blight of rice, *Xanthomonas oryzae* pv. *oryzae* [20], the well-known causal agent of the fungal take-all disease of wheat, *Gaeumannomyces graminis* var. *tritici* [21] and the oomycetes *Phytophthora* spp. and *Pythium* spp. [22–24]. PCA displays redox activity and is a nitrated heterocyclic antibiotic compound, notably due to its interference with the electron transport chain [25]. PCA has also been linked to biofilm formation, favoring attachment of PCA-producing *Pseudomonas* spp. to plant roots [25].

Recently, it has been demonstrated that PCA-producing *P. fluorescens* strain LBUM223 (hereafter named LBUM223) can alter the expression of key virulence genes in the bacterial potato pathogen *Streptomyces scabies*, reducing common scab disease symptoms [26, 27]. This effect is directly linked to PCA production since an isogenic *phzC-* mutant of LBUM223 incapable of producing PCA (hereafter named LBUM223*phzC*-), loses its biocontrol activity [27]. Interestingly, in this system PCA does not contribute to biocontrol activity by reducing the pathogen’s soil populations through toxicity. Instead, it was demonstrated that sub-inhibitory concentrations of PCA found in soil alter the transcriptome of *S. scabies*, which contribute to reducing virulence and disease symptom development [28, 29].

As PCA production by LBUM223 has been demonstrated as a key biocontrol determinant of common scab of potato through targeted transcriptomic changes in the pathogen, and that preliminary results obtained in our laboratory indicated that LBUM223 showed strong potential for repressing the growth of *P. infestans*, we proposed in this study to characterize the effect of PCA production by LBUM223 on the transcriptome of *P. infestans*. The availability of a reference genome for *P. infestans* allows the comparison of its expression profile obtained by performing RNA sequencing (RNA-Seq) analysis during a time course experiment where the pathogen is confronted with LBUM223, its isogenic *phzC*- mutant incapable of producing PCA, and synthetic PCA. This allows the identification of genes that are differentially expressed due to the presence of LBUM223 and/or its production of PCA, which might play a key role in the interaction between both organisms. To our knowledge, this is the first study investigating the impact of a potential biocontrol agent on the transcriptome of *P. infestans*. This study will provide a better understanding of the molecular mechanisms involved in this interaction, which might in turn contribute to developing and optimizing a biocontrol strategy against late blight of potato.

## Results

### *P. infestans*’ growth inhibition on in vitro plate assays

**Fig. 1:**
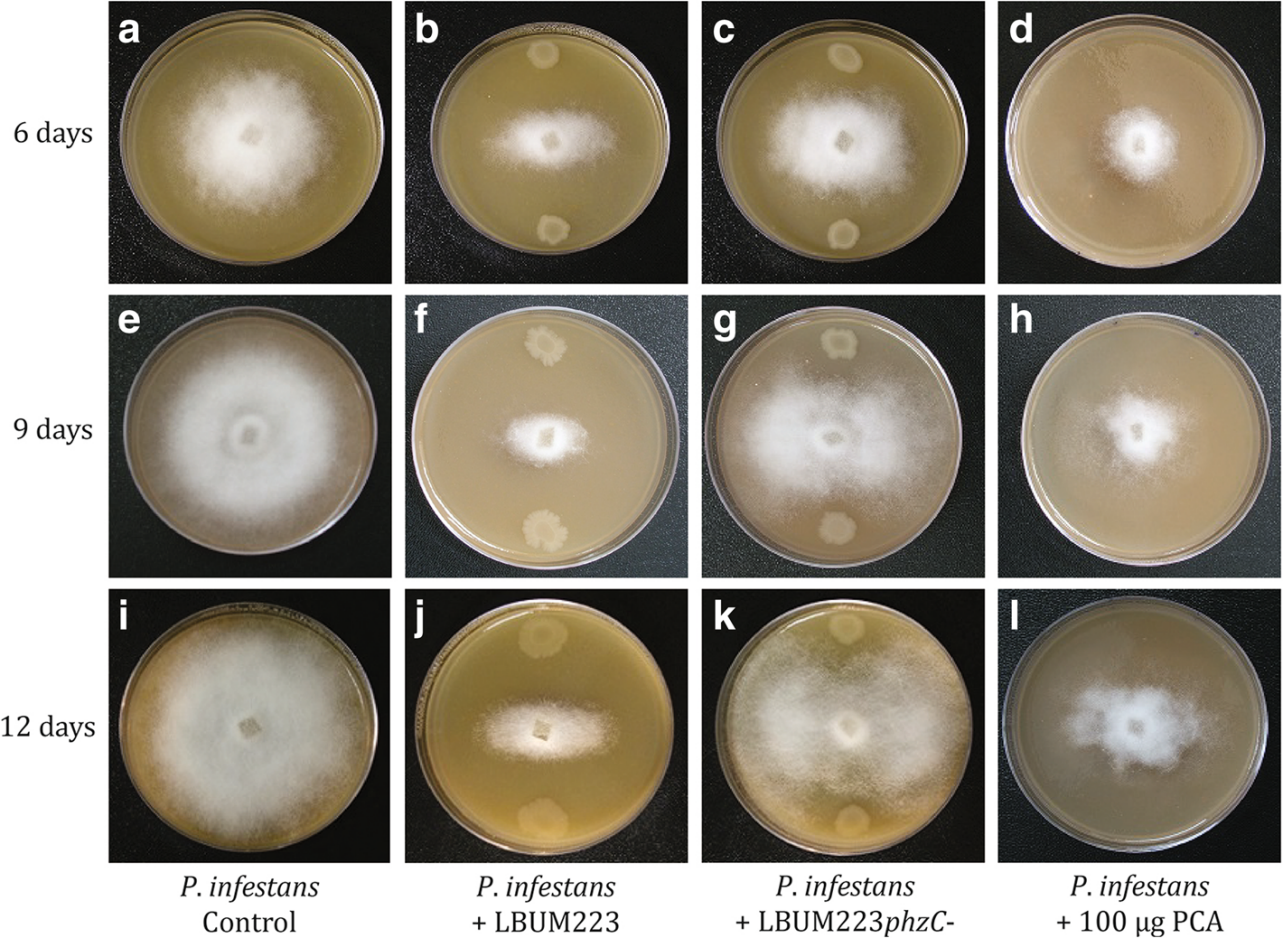
Confrontational plate assays of *P. infestans* treated with no antagonist (control; **a, e, i**), LBUM223 (**b, f, j**), LBUM223*phzC*- (**c, g, k**) and exogenically applied PCA (**d, h, l**) during a time course study at day 6 (**a, b, c, d**), day 9 (**e, f, g, h**) and day 12 (**i, j, k, l**)

**Fig. 2:**
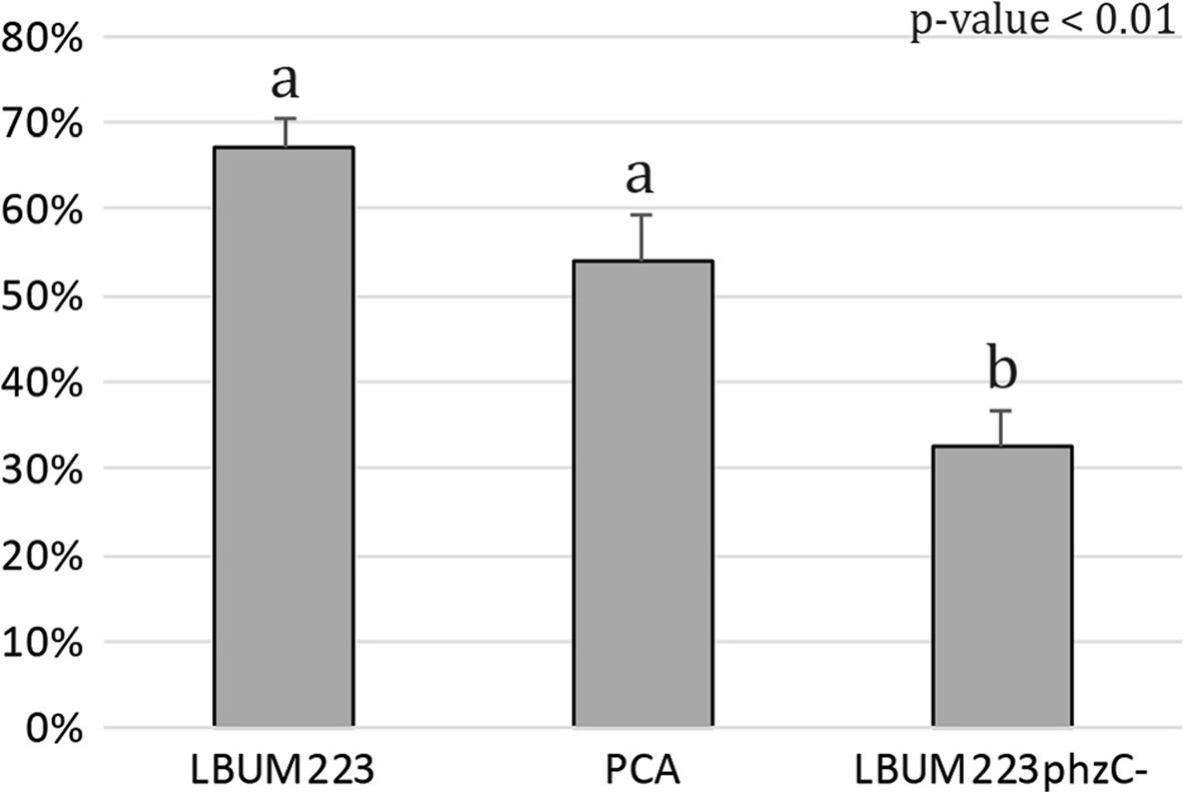
Effects of treatments on *P. infestans*’ percent growth inhibition. 100% represents complete growth inhibition while 0% represents no inhibition when compared to the control treatment. *P. infestans*’ growth inhibition in the presence of PCA (either produced by LBUM223 or exogenically applied) was significantly different than in the presence of LBUM223*phzC*- (*p*-value < 0.01)

Compared to the control (Fig. 1a, e, i), LBUM223 repressed the growth of *P. infestans* at 6, 9 and 12 days (Fig. 1b, f, j). PCA production by LBUM223 was clearly involved in *P. infestans’* growth inhibition, since the non-PCA producing LBUM223*phzC*- did not repress growth as much as LBUM223 (Fig. 1c, g, k). Synthetic PCA repressed *P. infestans’* growth similarly to LBUM223 (Fig. 1d, h, l). At 9 days, LBUM223 and exogenically applied PCA led to 67 and 54% growth inhibition rates compared to the control, respectively. These values are significantly different than the 32% growth inhibition rate obtained when *P. infestans* was co-inculated with LBUM223*phzC*- (Fig. 2).

### RNA-Seq data analysis

RNA sequencing was performed on RNA extracted from *P. infestans’* mycelium. For each time and each treatment, three biological replicates were sequenced. The total number of reads for each sample ranged from 42 to 85 million reads with a median of 82 million reads (Additional file 1). Data were filtered in order to exclude genes presenting less than 100 mapped reads when comparing the highest and the lowest expression values over all the samples and to retain data that was significantly differentially expressed with a false discovery rate (FDR)-corrected *p*-value < 0.05. Thus, for each treatment at day 6, day 9 and day 12, transcriptome profiles encompassed 12,513, 12,530 and 12,730 genes respectively on the 17,797 identified *P. infestans* genes (data not shown).

**Fig. 3:**
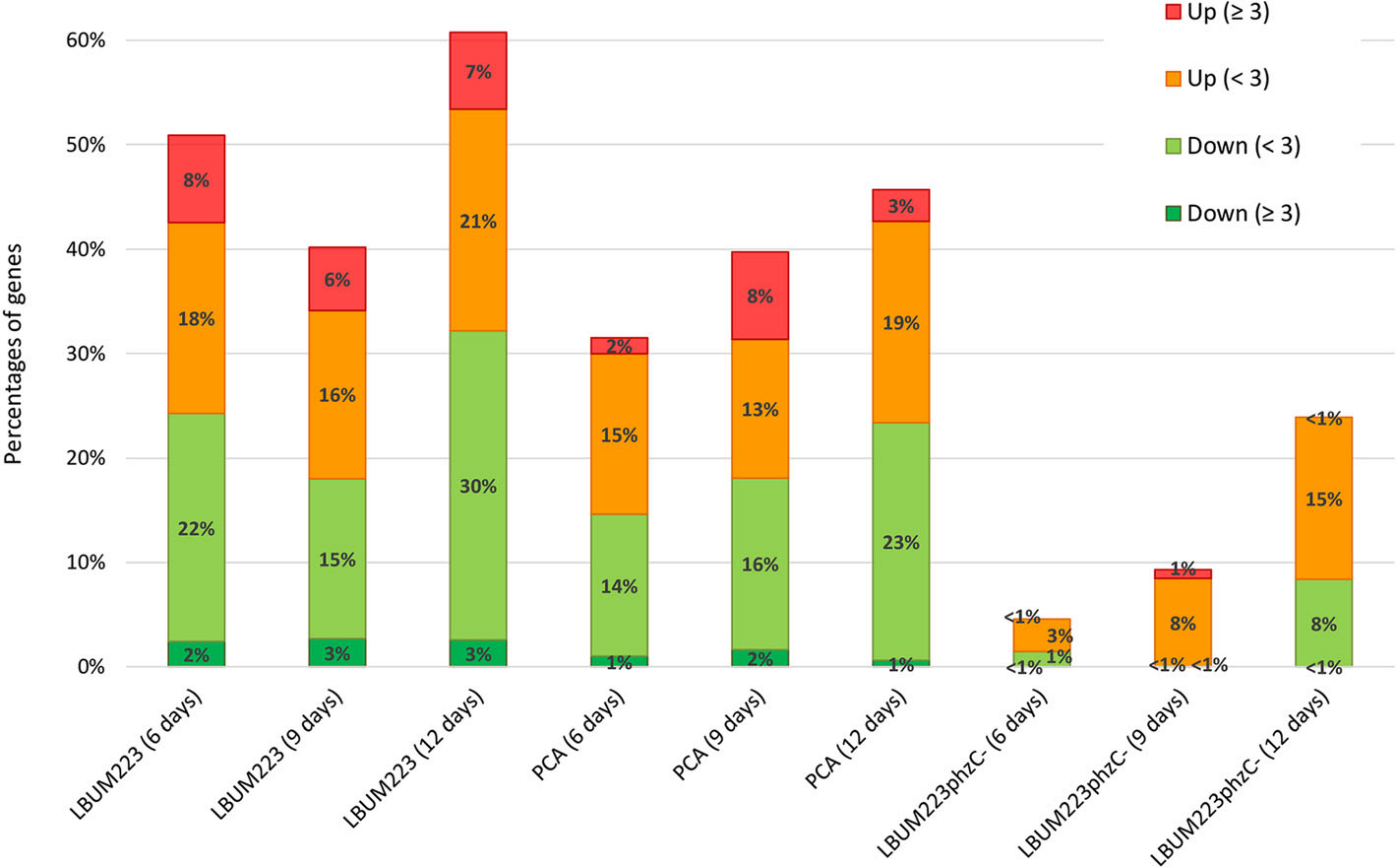
Distribution of fold changes expression at each time point by comparing each treatment to control. Gene expressions (FDR-corrected *p*-value < 0.05) were divided into 4 groups: Down-regulated with ≥ absolute 3 fold change (dark green); Down-regulated with < absolute 3 fold change (green); Up-regulated with < absolute 3 fold change (orange): Up-regulated with ≥ absolute 3 fold change (red). Down-regulated and up-regulated categories with < absolute 3 fold change (green and orange) exclude genes with absolute fold change values comprised between − 1 and 1

By comparing transcriptome profiles from each treatment to the control, the distribution of fold change expression was determined at each time point (Fig. 3). In accordance with the growth inhibition time course plate assay results (Fig. 1), the proportion of genes significantly altered in their expression (FDR-corrected p-value < 0.05) increased as time progressed (Fig. 3). At day 6 and 9, *P. infestans*’ growth was only slightly repressed by LBUM223*phzC*- and only 4 and 9% of genes were differentially expressed in *P. infestans*, respectively. At day 12, the growth inhibition was more important and 23% of *P. infestans’* transcriptome was altered by LBUM223*phzC*-. LBUM223 and PCA treatments led to more significant transcriptional alteration. At day 6, half (50%) of *P. infestans*’ transcriptome was altered by LBUM223 and 32% was altered by synthetic PCA. At day 9, both treatments altered 40% of *P. infestans*’ transcriptome. At day 12, more than 60 and 45% of *P. infestans*’ transcriptome was altered by LBUM223 and synthetic PCA, respectively. LBUM223 generally altered the expression of genes in *P. infestans* more importantly than exogenically applied PCA.

### Analyses of the differentially expressed genes

**Fig. 4:**
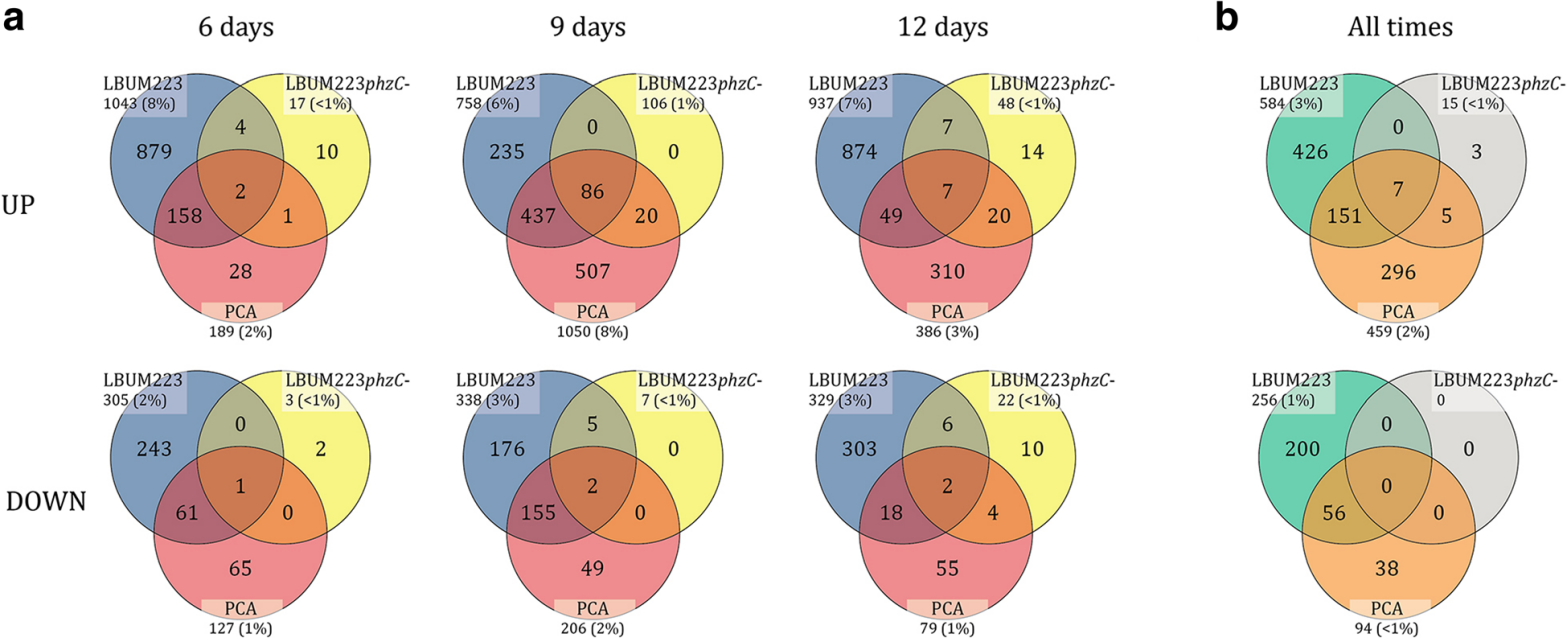
Numbers of DEGs in common among treatments. Comparison of significantly (**a**) up- and down-regulated genes common to *P. infestans* at three different time points (day 6, 9, 12) or (**b**) when all times are combined, when confronted to LBUM223, exogenically applied PCA and LBUM223*phzC*- (≥ absolute 3 fold change, FDR-corrected *p*-value < 0.05)

To analyse differentially expressed genes, an absolute fold change of 3 or more (either up- or down-regulated) was chosen as a cut-off. Genes significantly altered in their expression by PCA (either produced by LBUM223 or exogenically applied) were identified for each time point under study (Fig. 4a). 158, 437 and 49 up-regulated genes were shared between both treatments at day 6, 9 and 12, respectively, whereas 61, 155 and 18 genes were down-regulated at the same time-points. In general, there were close to 3 times more up-regulated than down-regulated genes. Also, it was observed that the number of up- or down-regulated genes was much higher at day 9 than the two other time points.

**Fig. 5:**
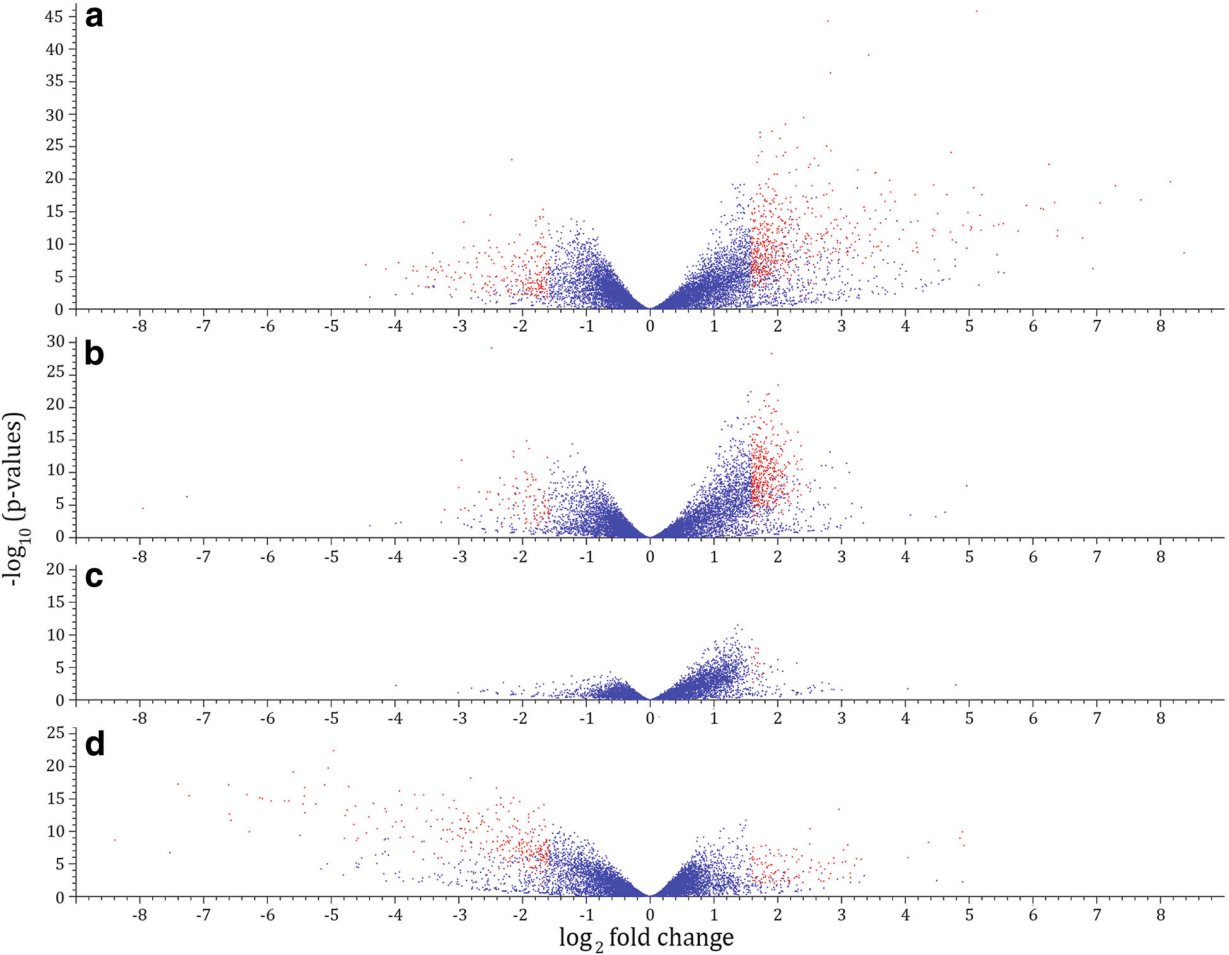
Volcano plots of differential gene expression in *P. infestans* when all time combined, comparing (**a**) control vs treatment with LBUM223 (**b**) control vs treatment with exogenically applied PCA (**c**) control vs treatment with LBUM223*phzC*- and (**d**) treatment with LBUM223*phzC*- vs treatment with LBUM223. Dots in red show genes that are significantly up- or down-regulated (≥ absolute 3 fold change). Plots were generated using the CLC Genomics Workbench software version 10.9.5

When combining all time points and performing the same analysis again, 151 up-regulated genes were shared between LBUM223 and exogenically applied PCA and 56 down-regulated genes were shared between these two treatments (Fig. 4b); the up-regulated genes being again almost three times more prevalent than the down-regulated ones. Differential gene expression data showed that *P. infestans* seemed to respond quite similarly to LBUM223 and exogenically applied PCA (Fig. 5a, b) with 840 (4.7%) and 553 (3.1%) differentially expressed genes, respectively. *P. infestans*’ transcriptome was almost not altered by exposure to LBUM223*phzC*- (Fig. 5c), with only 15 differentially expressed genes, representing less than 0.001% of the *P. infestans’* genome. 425 genes (2.4%) were differentially expressed when comparing *P. infestans* exposed to LBUM223 and to LBUM223*phzC*- (Fig. 5d).

**Fig. 6:**
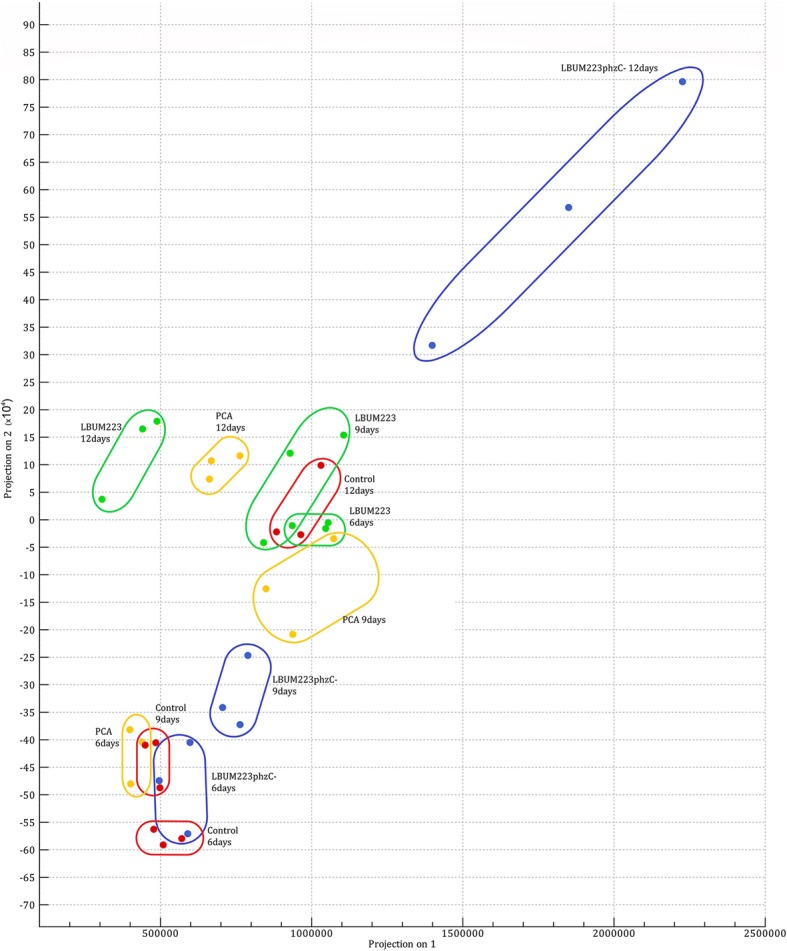
Principal component analysis of variability observed across samples. Expression values associated with each samples were spanned onto a two-dimensional plot. Treatments are grouped by treatment and time. Colors were used to differentiate between treatments: red for Control samples, blue for LBUM223phzC- samples, green for LBUM223 samples and yellow for PCA samples

Variability across samples for expression values was studied using principal component analysis. Samples were grouped by timepoint, replicate and treatment (Fig. 6). In general, replicate samples belonging to a given treatment and a given time clustered more closely together than with samples belonging to other treatments or time. LBUM223*phzC*- samples at day 12 displayed the most important variability between replicates. Some overlaps were detected between treatments and time, but overall, except for the control samples at day 12 and PCA samples at day 6, LBUM223 and PCA samples clustered closer together (irrespective of time) than with control or LBUM223*phzC*- samples (Fig. 6).

### Identification of the differentially expressed genes through time course analysis

**Fig. 7:**
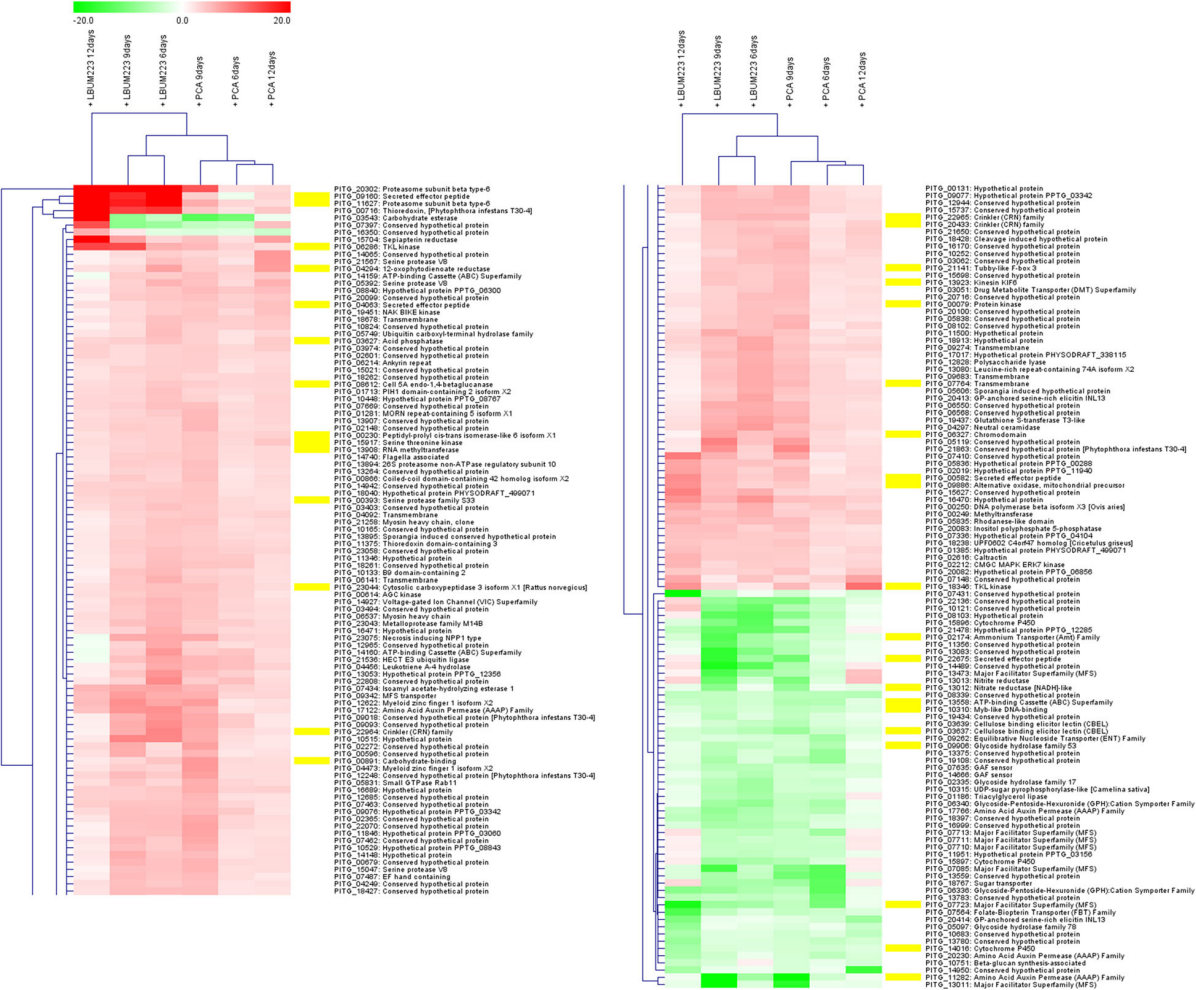
Hierarchical clustering according to changes in the expression of selected genes through time when *P. infestans* is confronted to LBUM223 or exogenically applied PCA at day 6, 9, 12. Cluster analysis was performed with the MEV program with Euclidean distance and average linkage. Yellow boxes are indicating genes for which expression was validated by RT-qPCR

The 207 genes either up- or down regulated by exposure to PCA (either produced by LBUM223 or exogenically applied) were identified using NCBI’s BLASTn algorithm and a hierarchical clustering was prepared for the different time points (Fig. 7). Almost half of the genes were characterized as encoding “hypothetical proteins”. Transcriptional changes appeared quite similar between all LBUM223 and PCA samples, except for the first four genes presented: subunits of the proteasome (PITG_20302 and PITG_11627), secreted effector peptide (PITG_09160) and thioredoxin (PITG_00716), which were clearly more up-regulated by exposure to LBUM223 than exogenically applied PCA. Only two genes, in addition to the 207 being significantly up- or down-regulated at all times, presented an opposite expression pattern: PITG_03543 and PITG_16350, being up-regulated by LBUM223 at day 12 but down-regulated by PCA at all times. After looking at all RXLRs and CRN encoding genes at each time and for all treatments with the absolute value of 3 fold change or more (data nor shown), it appeared that all differentially expressed CRN genes (PITG_22964, PITG_22965 and PITG_20433) were up-regulated, while most but not all differentially expressed RXLR genes (PITG_09160, PITG_00582, PITG_04063, but not PITG_22675) were also up regulated (at all time points).

### Functional classification of the differentially expressed genes

**Fig. 8:**
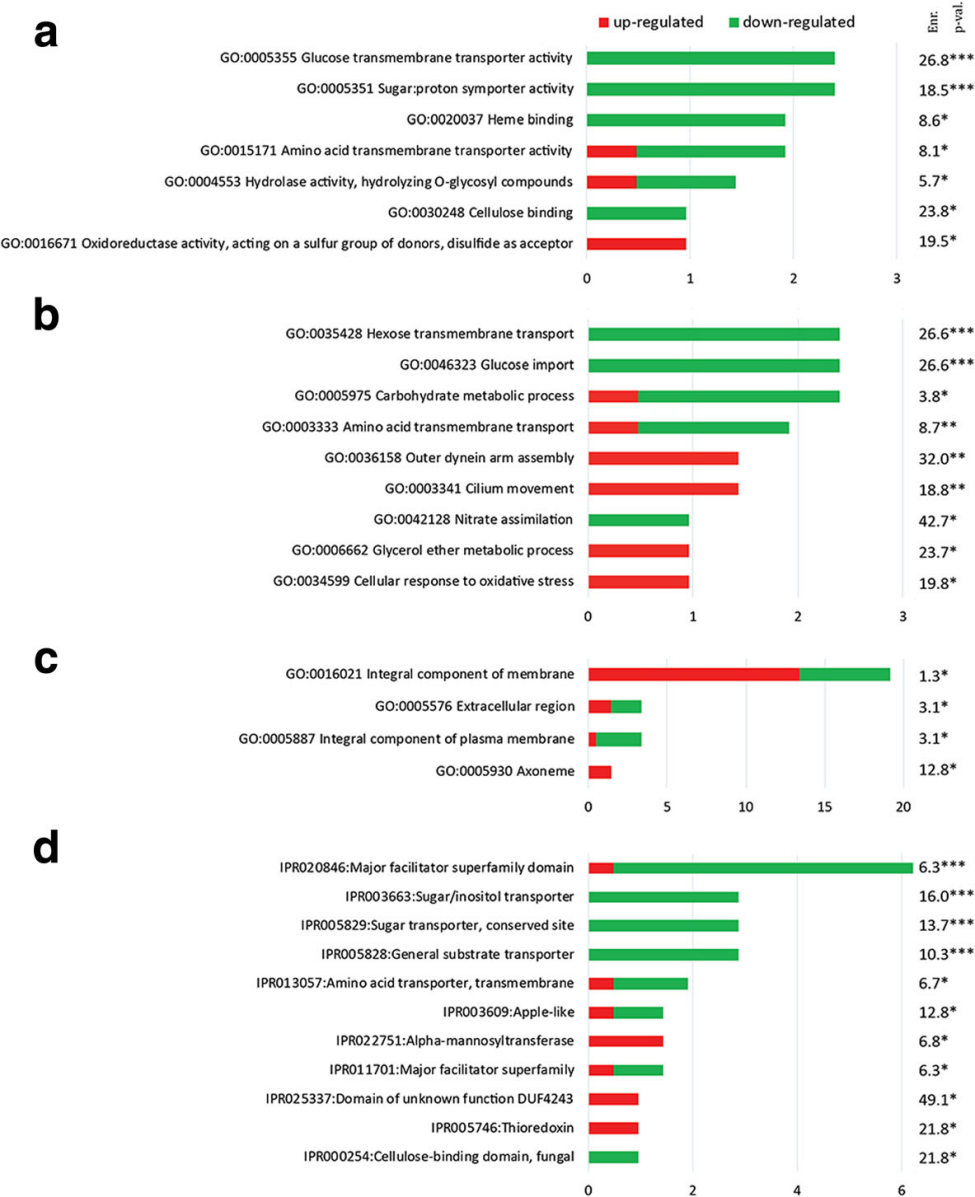
Gene ontologies and Interpro families enriched in *P. infestans*. Percentage of genes associated with enriched GO molecular function (**a**), GO biological process (**b**), GO cellular component (**c**) and protein family (**d**). Enrichment is abbreviated with Enr. and p-val represents the corresponding p-value (***, *p*-value < 0.001, **, *p*-value < 0.01, *, *p*-value < 0.1). Proportions of up-regulated and down-regulated genes are indicated with red and green colors, respectively

Gene Ontology (GO) analysis was performed and GO-terms were obtained using Blast2GO [30]. A GO enrichment analysis using the open web software DAVID [31] was also performed. Functional categories were ordered by percentages of gene involved, and then by enrichment values (Fig. 8). 7 “molecular function” ontologies, 9 “biological process” ontologies, 4 “cellular component” ontologies and 11 protein families were found enriched in *P. infestans*’ transcriptome (Fig. 8).

Among molecular functions (Fig. 8a), all enriched GO-terms were mostly associated with down-regulated-genes, except for GO:0016671 associated with oxidoreductase activity, representing 1% of up-regulated genes (PITG_000716 and PITG_11375 annotated as thioredoxins). GO:0005355 associated with glucose transmembrane transporter activity was the most enriched function with 2.4% of down-regulated cognate sequences. Other enriched GO-terms included: sugar, proton symporter activity (GO:0005351), heme binding (GO:0020037), amino acid transmembrane transporter activity (GO:0015171), hydrolase activity (GO:0004553) and cellulose binding (GO:0030248).

Among biological processes (Fig. 8b), GO:0035428 associated with hexose transmembrane transporter activity, GO:0046323 associated with glucose import and GO:0005975 associated with carbohydrate metabolic process were the most represented in percentages, each representing 2.4% of associated sequences. Most of the genes associated with these GO-terms were down-regulated. The most enriched process was however GO:004128 associated with nitrate assimilation, for which differentially expressed genes were only down-regulated. Other enriched GO-terms included: amino acid transmembrane transport (GO:0003333), outer dynein arm assembly (GO:0036158), cilium movement (GO:0003341), glycerol ether metabolic process (GO:0006662) and cellular response to oxidative stress (GO:0034599).

For cellular component (Fig. 8c), GO:0005930 associated with axoneme was the most enriched, although representing only 1.4% of differentially expressed genes, all being up-regulated genes. GO:0016021 associated with integral component of membrane was represented by 19.6% of differentially expressed gene sequences. Other enriched GO-terms included: extracellular region (GO:0005576) and integral component of plasma membrane (GO:0005887).

More than 6% of gene sequences, mostly down-regulated, encode a domain belonging to the major facilitator protein family (IPR:020846) (Fig. 8d). Other protein families for which gene expression was mostly down-regulated involve transporters for sugar/inositol (IPR:003663, IPR:005829) and general substrate transporter (IPR:005828). The most enriched protein category however involves a domain of unknown function (IPR:025337), followed by thioredoxin (IPR:005746) and cellulose-binding (IPR:000254). Other enriched protein families included: amino acid transporter (IPR:013057), apple-like domain (IPR:003609), alpha-mannosyl transferase (IPR022751), and major facilitator superfamily (IPR:011701).

To complement the list of functional categories altered in *P. infestans* by PCA (produced by LBUM223 and exogenically applied) a KEGG analysis was performed, highlighting information about the metabolic pathways associated with the 207 genes altered in their expression (Additional file 2). As more genes were up- than down-regulated, it was not surprising to detect more metabolic pathways associated with up-regulated gene expression. The two most represented metabolic pathways in this analysis were purine and thiamine metabolisms, with 23 and 21 genes sequences, respectively, associated with adenylpyrophosphatases (ec:3.6.1.3), phosphatases (ec:3.6.1.15), hydrolases (ec:3.5.2.17) and kinases (ec:2.7.1.20) (data not shown). The nitrogen-associated and phenylalanine, tyrosine and tryptophan biosynthesis metabolic pathways were the only enriched pathways strictly associated with down-regulated genes.

### Validation of differentially expressed genes

**Fig. 9:**
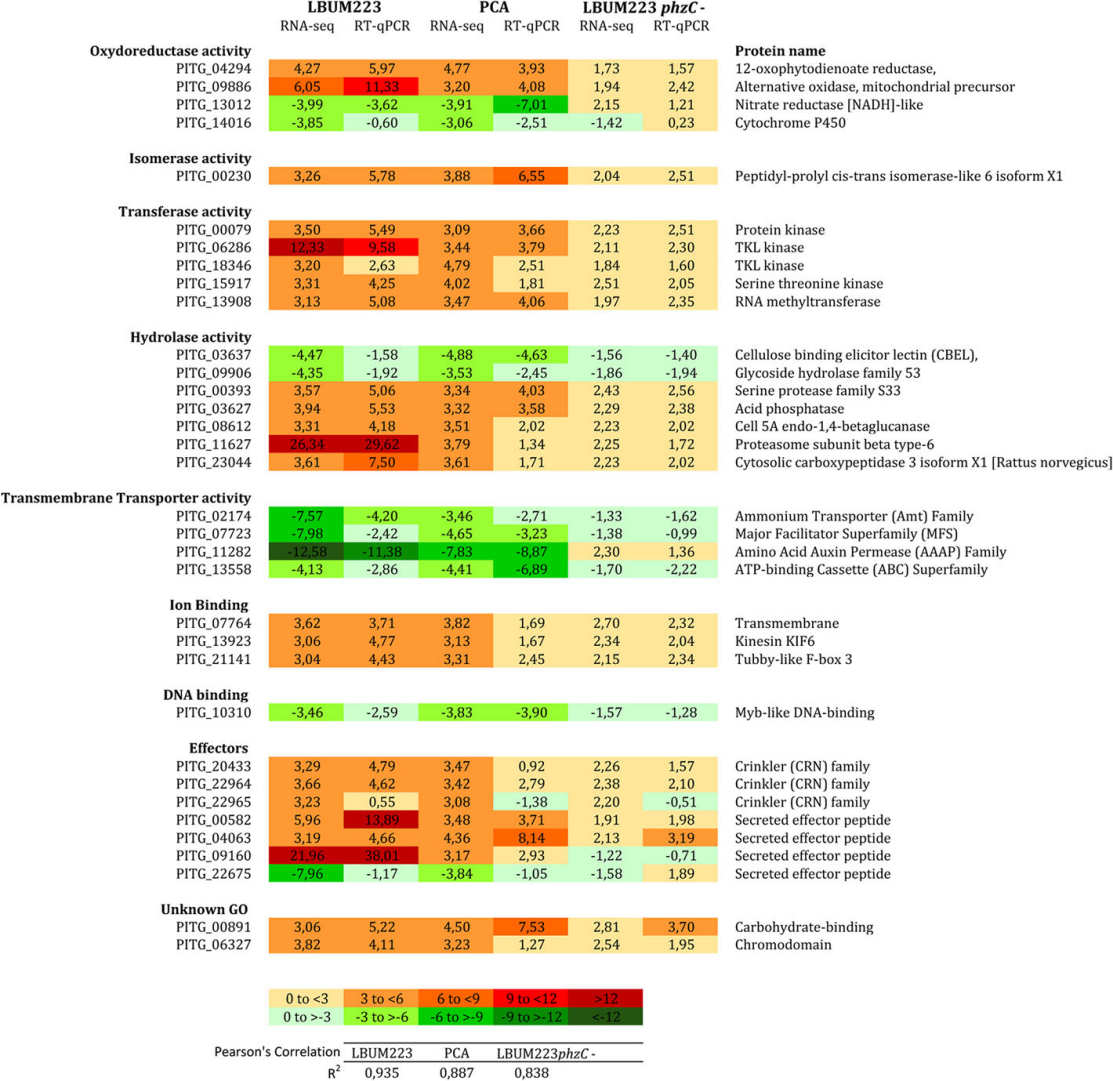
Selected target genes validated by RT-qPCR. Difference in fold change for gene expression in *P. infestans* between different treatments: in the presence of LBUM223, exogenically applied PCA or LBUM223*phzC*-. For each treatment, differences in gene expression were obtained by RNA-Seq (all times combined) and RT-qPCR. For RT-qPCR, the fold changes represent differential gene expressions observed between treatments and control. Shades of red are associated with up-regulation of gene expression, while shades of green were used for down-regulation of gene expression. Pearson’s correlations were calculated for each treatment between RNA-Seq and RT-qPCR results (*p* < 0.0001): R^2^_(LBUM223)_ = 0.935, R^2^_(PCA)_ = 0.887 and R^2^_(LBUM223*phzC*-)_ = 0.838

From the list of 207 genes differentially expressed genes with a significant fold change of at least 3 (either up- or downregulated) when all time points were combined, a selection of 34 differentially expressed genes (Fig. 7: yellow marks) was made to reflect a diversity of functional categories. The category “unknown GO” represented sequences which did not receive a cognate “molecular function” GO-term. For these genes, the results obtained by RNA-Seq were compared to those obtained by RT-qPCR. Almost all the up-regulated genes identified using RNA-Seq were also found to be up-regulated by RT-qPCR. The same was also observed for the down-regulated genes (Fig. 9). A Pearson’s correlation gave 93.5% of similarity between RNA-Seq and RT-qPCR results when *P. infestans* was confronted with PCA produced by LBUM223 and 88.7% for exogenically applied PCA (Fig. 9).

## Discussion

The results obtained in this study not only showed that phenazine-1-carboxylic acid (PCA) produced by *Pseudomonas fluorescens* strain LBUM223 inhibited *Phytophthora infestans’* growth, but also suggest that PCA significantly altered gene expression in *P. infestans* over time.

PCA is known as an important broad-spectrum antimicrobial compound involved in the biocontrol of various plant pathogens, including *Phytophthora* spp. [22, 24, 32]. Generally, due to its nitrogen-containing heterocyclic composition, PCA is mainly described as promoting redox reactions and generating reactive oxygen species (ROS), of which the accumulation leads to a reduction in energy production, carbohydrate metabolism, and nutrient uptake [33, 34]. The capacity of PCA to specifically alter the expression of key genes involved in biological functions of interest in *P. infestans* has however, to our knowledge, never been demonstrated before.

We first determined if housekeeping functions in *P. infestans* were altered in response to PCA, such as transcription (GO:0010467), translation (GO:0006412), structural constituents of ribosome (GO:0003735), metabolism (GO:0044237), and cellular metabolic process and regulation of catalytic activity (GO:0050790), as identified in [35]. Only three gene sequences among the 207 genes identified in our study were associated with two housekeeping functions: GO:0006412 and GO:0003735 (data not shown). These were all up-regulated and identified as an adenine nucleotide transporter (PITG_13053) and two belonging to the mitochondrial carrier family (PITG_18261 and PITG_18262). This suggest that PCA does not alter the basal metabolism in *P. infestans*, but rather mainly alters its secondary metabolism.

Among *P. infestans’* functions altered in response to PCA exposure, either being produced by LBUM223 or exogenically applied, oxidoreduction, transport and phosphorylation were the most altered, with associated genes being either up- or down-regulated. Among them, three cytochrome P450-coding genes (PITG_15896, PITG_15897 and PITG_14016; Figs. 7 and 9) were down-regulated, suggesting that the cytochrome respiratory pathway is restricted and explaining why an alternative oxidase-coding gene (PITG_09886) is up-regulated (Fig. 9). Alternative oxidase proteins are known to be localized in the mitochondria and may increase respiration when the cytochrome respiratory pathway is restricted [36]. Also, the nitrogen metabolism pathway represented by the nitrate assimilation cluster (PITG_13011, PITG_13012, and PITG_13013 corresponding to the major facilitator superfamily transporter also known as nitrate transporter, nitrate reductase, and nitrite reductase, respectively) was down-regulated in response to PCA exposure (Figs. 7, 8, 9). These reductase proteins convert nitrate to nitrite to ammonium, to liberate nitrogen as a source for amino acids synthesis, especially for the glutamine/glutamate synthetase pathways [37]. A down regulation of this cluster most likely leads to less available nitrogen for amino acid synthesis. A recent study also demonstrated that silenced strains of *P. infestans* for the nitrate assimilation gene cluster could still infect potato tubers but became non-pathogenic on leaves [37]. Three nitrate transporters, including PITG_13011, did not show much transcriptional activity in a previous study performed under in vitro conditions during the hyphae stage [12]. Thus, the down-regulation of this nitrate cluster observed in our study under in vitro conditions seems quite new in the presence of PCA.

Our results also showed that transport functions represented by amino acid transporters, glucose transmembrane transporters and major facilitator family proteins were mainly down-regulated (Figs. 7, 8 and 9). These include an ammonium transporter (PITG_02174) and an ATP-binding cassette (ABC) transporter (PITG_13558), suggesting a decreased ability for *P. infestans* to efficiently uptake some nutrients, including nitrate (PITG_13011). Moreover, ABC transporters have been shown to contribute to fungicide resistance, as deletion of four ABC transporter encoding genes in *Fusarium* sp. increased its sensitivity to some fungicide [38]. These results are contrary to another study where one-third of amino acid transporters were expressed mostly in hyphae, compared to other life stages [12]. As PCA contains a redox active nitrogen heterocycle, these changes in gene expression could be part of a defense mechanism for *P. infestans* to protect itself by avoiding PCA import, using a yet uncharacterized mechanism. This hypothesis would however need to be further validated.

Other *P. infestans’* functions altered in response to PCA exposure, whether produced by LBUM223 or exogenically applied, are Kinase (GO:0016301) and phosphatase (GO:0016791) activities, which were altered with numerous up-regulated genes (Figs. 7 and 9), including genes encoding TKL (tyrosine kinase-like) kinases (PITG_06286 and PITG_18346) and acid phosphatases (PITG_03627). Phosphorylation mechanisms have been reviewed as involved in CRN virulence function [39]. To date, it is not known which kinases might be responsible for the required CRN phosphorylation and their putative secretion, delivery and stability [39]. Based on the results obtained, serine threonine kinases and tyrosine kinases could possibly participate in the regulation of CRN and RXLR effectors, which however remains to be further investigated. This is in agreement with the identification of members of large effector families whose phosphorylation impacts host-pathogen interactions [40], including roles in *Phytophthora* spp. life cycles [41].

RXLR effectors-coding genes were overall more up-regulated than down-regulated (Fig. 9), while CRN effectors-coding genes appeared to be only up-regulated (Fig. 9). Since our study was performed under in vitro conditions, it is surprising to observe such an up-regulation of numerous effectors, notably CRN genes, without the presence of the plant normally responsible for their induction. An overexpression of effectors encoding genes was also reported as unexpected in an in vitro plate assay looking at the acquired resistance to the chemical fungicide mefenoxam in sensitive isolates of *P. infestans*. Interestingly, PITG_09160 expression was upregulated in both studies [10]. This is contrary to what was observed in previous published studies following plant infection by the pathogen [1, 7, 8]. At early stages following infection, up-regulation of effector encoding genes is expected, presumably to suppress PTI (PAMP (pathogen associated molecular patterns)-triggered immunity). Effectors can also trigger ETI (effector-triggered Immunity) in plants possessing the corresponding R gene [42]. Plants are known to produce reactive oxygen species as part of their defense responses [43], and therefore it is possible that *P. infestans* reacts to PCA, similarly as if a plant was present. CRN effectors phosphorylation studies also suggested that CRN proteins could be involved in other functions than causing crinkling and necrosis [40]. In this study, we postulate that LBUM223, via its production of PCA, causes a metabolic disturbance in *P. infestans*, leading to the overexpression of several effectors-coding genes even in the absence of the plant, which may play an active role in biocontrol.

Interestingly, four genes (PITG_20302, PITG_09160, PITG_11627 and PITG_00716) were more up-regulated in the presence of LBUM223 than in the presence of exogenically applied PCA (Fig. 7). An explanation could be the recognition by *P. infestans* of different surface molecules or even some secreted proteins of LBUM223. After all, up to 23% of the *P. infestans*’ transcriptome is also altered by exposure to the isogenic mutant not-producing PCA (Fig. 3). Lipopolysaccharides (LPS), which are bacterial cell wall components, have been shown to have a variety of implications, including triggering induction of ROS, similarly to PCA [34, 44]. It was previously demonstrated that LPS induces an oxidative burst in the brown alga *Laminaria digitate* [44], which also belong to the Stramenopiles like *P. infestans*. More specifically, ROS production oxides a thioredoxin protein (Trx), which is known to regulate kinases, like the apoptosis signal-regulating kinase 1 (ASK1) [45] involved in the suppression of cell death [46]. The activated complex ASK1/Trx can however lead to cell death [45]. Although often described in mammalian cells, Trx remains a highly-conserved protein. In our results, we observed that two thioredoxin coding genes are up-regulated (PITG_00716 and PITG_11375) and one notably by LBUM223 (Figs. 7 and 9). When life stages were compared, another study also showed that ASK1 is rapidly ubiquitinated and degraded when activated by ROS [47]. One of the ubiquitin has been identified as an E3 ubiquitin ligase and conferred resistance to infection by *Pseudomonas aeruginosa* [47]. Here, we observed an over-expression of the ubiquitin protein-related family coding genes (PITG_21536: HECT E3 ubiquitin ligase and PITG_05749: ubiquitin carboxyl-terminal hydrolase family) and of the proteasome subunits coding genes (PITG_20302, PITG_11627, and PITG_13894) (Figs. 7, 9). Thus, we are suggesting that *P. infestans* is avoiding cell death by producing more thioredoxin proteins, and ubiquitinating and degrading ASK1, compensating the ROS-activation ASK1/Trx complex.

## Conclusion

Our study suggests that PCA is involved in *P. infestans’* growth repression and led to important transcriptomic changes by both up- and down-regulating gene expression in *P. infestans* over time. These differentially expressed genes are involved in many functions, including transmembrane transport activity, oxidoreduction activity and phosphorylation mechanisms, and includes effector genes. The genes differentially expressed in response to PCA exposure represent good candidates to be further studied during plant infection, as they might lead to a better understanding of the mechanisms involved in biocontrol of late blight by LBUM223.

## Methods

### Microorganisms and growth conditions

*Phytophthora infestans* US-8 was maintained on V8 medium agar plates at 20 °C. *Pseudomonas fluorescens* strain LBUM223 was isolated from the rhizosphere of strawberry plants in Bouctouche, NB, Canada and its genome was recently sequenced [48]. *Pseudomonas fluorescens* strain LBUM223*phzC-* is an isogenic mutant of LBUM223, incapable of producing PCA that was previously described and validated [27]. The LBUM223 and LBUM223*phzC*^*−*^ strains were routinely grown in tryptic soy broth (BD, Franklin Lakes, NJ) under constant agitation at 28 °C.

### Confrontational plate assays

All confrontational plate assays were carried out in Petri plates containing 20 mL of 10% V8 unclarified medium [49]. Three sampling dates (6, 9 and 12 days post-co-inoculation) were chosen to cover the time period occurring between initial interaction between *P. infestans* and LBUM223 and prior to reduced viability starting to occur 15 days post-inoculation (data not shown). Preliminary tests showed that mycelium destructive sampling before 6 dpi and after 12 dpi did not provide enough RNA to perform RNA-Seq analyses. Furthermore, at 6dpi, both organisms were too distant on the plate to significantly interact together and therefore harvesting prior to 6 dpi would have probably led to detecting no significant transcriptomic changes. For each of the three different sampling dates, the following treatments were prepared in triplicate, generating 36 samples in total: (1) *P. infestans* (no antagonist; control), (2) *P. infestans* + LBUM223, (3) *P. infestans* + LBUM223*phzC*-, (4) *P. infestans* + 100 μg PCA. 5 mm^2^ square plugs of *P. infestans* were placed in the center of each Petri plate. *Pseudomonas* spp. populations were determined by measuring absorbance (600 nm) (and referring to standard curves made by using serial dilutions of LBUM223 and LBUM223*phzC*^*−*^ in TSB). Both the LBUM223 and LBUM223*phzC*- cultures averaged 2 × 10^9^ CFU/mL. 20 μL of the appropriate *Pseudomonas* spp. culture or 50 μg of purified PCA (InFarmatik, Newark, DE, dissolved in anhydrous ethanol (0.5 μg/μL)), were spotted on two extremities of the plate and air-dried. Plates were incubated using a complete randomized block design at 20 °C during 6, 9 and 12 days (destructive sampling). At each harvesting time and for each treatment, *P. infestans*’ mycelium was manually collected using a spatula and immediately frozen in liquid nitrogen. For each sample, the mycelium was ground in liquid nitrogen using an RNAse-free mortar and pestle.

*P. infestans*’ inhibition growth rates were measured at day 9 using the method described by Daayf, et al. [50]. Statistical data analyses were performed using an ANOVA and Tukey’s posteriori tests using SAS University Edition 1 on Oracle VirtualBox.

### RNA extraction, DNAse treatments and quality control

RNA extractions were performed using the RNeasy Plant Mini Kit (Qiagen, Mississauga, Canada) as described by the manufacturer with the following modifications. Extractions were carried out using 100 mg of mycelium powder and the optional on-column DNase digestion with the RNase-Free DNase set was performed as described by the manufacturer. Each undiluted RNA extract was subjected to two consecutive rounds of DNAse treatment using a Turbo DNAse (TURBO DNA-free, Life Technologies, Carlsbad, CA, USA), as described by the manufacturer. Purified RNA was verified and quantified by using Experion StdSens chips (Bio-Rad, Mississauga, Canada) and stored at − 80 °C.

### RNA sequencing

A total of 36 samples (12 triplicate samples) were submitted to the McGill University and Genome Quebec Innovation Centre (Montreal, Québec, Canada) for sequencing. Prior to sequencing using the Illumina HiSeq 2000 Paired-End 100 bp, ribosomal RNA depletion, library preparation (mRNA stranded; using Illumina Truseq RNA adaptors), and quality assessment were performed.

### Availability of data and materials

The datasets generated and/or analysed during the current study are available in the NCBI SRA repository under the study accession number SRP119407. SRA records are accessible with the following link: https://www.ncbi.nlm.nih.gov//bioproject/PRJNA413149.

### RNA-Seq analysis

Data analyses were carried out using the CLC Genomics Workbench software version 9.0.1 (CLC bio, Boston, MA) [51]. Sequencing reads for each sample (Additional File 1) were mapped to *P. infestans* T30–4 reference genome (GenBank accession number AATU01000000) [1]. Expression values were calculated in total counts (giving for eAach gene the number of reads mapped to the exons of that gene and for each transcript the total number of reads mapped to the transcript).

### Identification of differentially expressed genes

The integrated EdgeR Bioconductor package [52] was used to determine differential gene expression by normalizing total transcript read counts using the trimmed mean of M-values method with default parameters and with a comparison between all pairs and the FDR-corrected *p*-value [53]. Transcript reads from biological replicates were grouped in 4 groups (*P. infestans* without confrontation = Control, *P. infestans* confronted with LBUM223, *P. infestans* confronted with LBUM223*phz*C-, *P. infestans* in the presence of PCA). This was done for each time (6, 9 and 12 days) but a general analysis was also performed for all time combined. For both analyses, data were filtered in order to exclude genes presenting less than 100 mapped reads and any residual ribosomal RNA and tRNA (which were included among gene-coding regions). This cut-off of 100 mapped reads represents the difference between the highest and the lowest expression values obtained over all samples. False discovery rate (FDR)-corrected *p*-values of less than 0.05 and fold changes greater than the absolute value of 3 were used as criteria for significant differentially expressed genes.

To proceed with the functional analysis, the same procedure as before was performed with all times and replicates combined as one treatment. Differential gene expression was then calculated from the mean of nine samples: gene expression of the three replicates at the three different times. Using the same filters, a list of 207 genes was obtained, these genes were either up- and/or down-regulated with an absolute value of fold change of 3 or more and a FDR-corrected p-value less than 0.05 for both treatments: LBUM223 and PCA. Thus, all these genes were only altered by PCA, either produced by LBUM223 or exogenically applied.

### Principal component analysis of variability observed across samples

A covariance matrix was generated for all samples from the cognate expression values using the “principal component analysis” option of CLC Genomics Workbench software version 9.0.1. All samples were spanned onto a two-dimensional space and coloured by treatment.

### GO-terms and pathway enrichments of differentially expressed genes

Blast2GO [30] was used to identify, map and annotate transcripts. CloudBLAST using a blastx algorithm and eukaryota parameters were used. The mapping was done with the default parameters, recovering the GO-terms, which are associated to the hits obtained by CloudBLAST. Selection of specific GO-terms from the GO pool obtained by the mapping and annotation steps was performed and assigned to the query sequences. Using the Kyoto Encyclopedia of Genes and Genomes (KEGG) function of Blast2GO, enzyme code (EC) annotations were provided from the GO annotation file. 44 gene sequences mapped to the KEGG metabolic pathway database. The KEGG map module allowed the identification of the metabolic pathways in which enzymatic functions participate. Functional enrichment analysis was carried out using the Database for Annotation, Visualization and Integrated Discovery (DAVID, version 6.8), using the default settings [31, 54, 55].

### HeatMap

From the identified genes list, data were downloaded into the open-source MultiExperiment Viewing (MEV) software version 4.9.0 [56, 57]. A heatmap was created from a hierarchical clustering with the following parameters: gene and sample trees selection with a gene and sample leaf order optimizations. Euclidean distance and average linkage clustering were selected for distance metric selection and linkage method selection, respectively.

### Reverse transcription - real time polymerase chain reaction (RT-qPCR)

To confirm RNA-Seq result, all 36 RNA samples were converted in cDNA using the TaqMan retrotranscriptase kit from Invitrogen. For each sample, 200 μL of cDNA was obtained for the following qPCR. 44 μL MgCL2 (25 mM), 40 μL dNTP (10 mM), 20 μL Buffer 10×, 10 μL Random Hexamers (50 μM), 4 μL RNAse inhibitor, 5 μL Reverse Transcriptase, 25.6 μL RNA and 51.4 μL H_2_O was used. The reverse transcription protocol was 10 min at 25 °C, then 30 min at 37 °C, 5 min at 95 °C and a final incubation at 4 °C. All cDNA samples were analysed by qPCR using either SYBR or TaqMan Technologies through Bio-Rad CFX manager software version 3.1. Additional File 3 lists all the primers used and designed with Primer Express 3.0.1. Some probes were also designed for a more specific detection of 7 targeted genes. 20 μL qPCR reactions were performed with primers and/or probes at a final concentration of 200 nM. 2 μL or 4.8 μL of cDNA were added to the SYBR or TaqMan mixes, respectively (iTaq Universal SYBR Green or Probes Supermix, Bio-Rad, Mississauga, Canada). For SYBR, the program consisted of 1 min at 95 °C, 10 s at 95 °C, 1 min at 60 °C for 45 cycles and a melt curve program was added with 5 s at 65 °C and a temperature gradation from 65 °C to 95 °C. For TaqMan, the program was 2 min at 95 °C, 5 s at 95 °C and 30 s at 60 °C for 45 cycles. Reference genes (encoding a subunit of the 40S ribosomal protein S3a and the ef1a elongation factor) were used and the expression values were normalized using qbase^PLUS^ software version 2.6.1 (Biogazelle, Zwijnaarde, Belgium). Pearson’s correlations were performed between RNA-Seq and RT-qPCR results (*p* < 0.0001) with SAS University Edition 1 on Oracle VirtualBox.

## Additional files

Additional file 1: Total for each sample of sequenced and mapped reads and the percentage of coverage quality. C for *P. infestans* without confrontation = control, B for *P. infestans* with LBUM223, M for *P. infestans* with the mutant LBUM223*phz*C- and N for *P. infestans* with synthetic PCA. 1 was used for day 6, 2 for day 9, 3 for day 12. X, Y, Z are for the 3 replicates. (XLSX 13 kb)

Additional file 2: Metabolic pathways associated with the 207 differentially expressed genes. A KEGG analysis performed using Blast2GO identified 36 metabolic pathways. For each metabolic pathway, the number of genes either up- or down-regulated, as well as the corresponding number of encoded enzymes were determined. (XLSX 10 kb)

Additional file 3: List of selected genes validated by qPCR. The expression of 27 targeted genes and 2 references genes was studied using the SYBR green technology, while the expression of 7 targeted genes was studied using the TaqMan probe technology. (XLSX 12 kb)

## Abbreviations

ABC transporter: ATP-binding cassette transporter
ASK1: Apoptosis signal-regulating kinase 1
CRN genes: Crinklers genes
DAVID: Database for Annotation, Visualization and Integrated Discovery
EC: Enzyme code
ETI: Effector-trigger immunity
FDR: False discovery rate
GO: Gene Ontology
KEGG: Kyoto Encyclopedia of Genes and Genomes
LBUM223: *Pseudomonas fluorescens* strain LBUM223
LBUM223*phzC*-: *Pseudomonas fluorescens* strain LBUM223*phzC*-
LPS: lipopolysaccharide
MEV: MultiExperiment Viewing
PAMP: Pathogen associated molecular patterns
PCA: Phenazine-1-carboxyllic acid
PDA: Potato dextrose agar
PTI: PAMP-triggered immunity
RNA-Seq: RNA sequencing
ROS: Reactive oxygen species
RT-qPCR: Reverse transcription real time polymerase chain reaction
Trx: Thioredoxin protein

## References

[1] Haas BJ, Kamoun S, Zody MC, Jiang RHY, Handsaker RE, Cano LM (2009). Genome sequence and analysis of the Irish potato famine pathogen *Phytophthora infestans*. Nature.

[2] Raffaele S, Win J, Cano LM, Kamoun S (2010). Analyses of genome architecture and gene expression reveal novel candidate virulence factors in the secretome of *Phytophthora infestans*. BMC Genomics.

[3] Kamoun S (2006). A catalogue of the effector secretome of plant pathogenic oomycetes. Annu Rev Phytopathol.

[4] Stassen JHM, Van den Ackerveken G (2011). How do oomycete effectors interfere with plant life?. Curr Opin Plant Biol.

[5] Kamoun S (2007). Groovy times: filamentous pathogen effectors revealed. Curr Opin Plant Biol.

[6] Schornack S, Huitema E, Cano LM, Bozkurt TO, Oliva R, van Damme M (2009). Ten things to know about oomycete effectors. Mol Plant Pathol.

[7] Pais M, Win J, Yoshida K, Etherington GJ, Cano LM, Raffaele S (2013). From pathogen genomes to host plant processes: the power of plant parasitic oomycetes. Genome Biol.

[8] Stam R, Jupe J, Howden AJ, Morris JA, Boevink PC, Hedley PE, Huitema E (2013). Identification and characterisation CRN effectors in *Phytophthora capsici* shows modularity and functional diversity. PLoS One.

[9] Fry WE (2008). *Phytophthora infestans*: the plant (and R gene) destroyer. Mol Plant Pathol.

[10] Childers R, Danies G, Myers K, Fei Z, Small IM, Fry WE (2015). Acquired resistance to mefenoxam in sensitive isolates of *Phytophthora infestans*. Phytopathology.

[11] Forbes GA (2012). Using host resistance to manage potato late blight with particular reference to developing countries. Potato Res.

[12] Ah-Fong AM, Kim KS, Judelson HS (2017). RNA-seq of life stages of the oomycete *Phytophthora infestans* reveals dynamic changes in metabolic, signal transduction, and pathogenesis genes and a major role for calcium signaling in development. BMC Genomics.

[13] Ah-Fong AM, Shrivastava J, Judelson HS (2017). Lifestyle, gene gain and loss, and transcriptional remodeling cause divergence in the transcriptomes of *Phytophthora infestans* and *Pythium ultimum* during potato tuber colonization. BMC Genomics.

[14] Divon HH, Fluhr R (2006). Nutrition acquisition strategies during fungal infection of plants. FEMS Microbiol Lett.

[15] Judelson HS, Tani S, Narayan RD (2009). Metabolic adaptation of *Phytophthora infestans* during growth on leaves, tubers and artificial media. Mol Plant Pathol.

[16] García-Bayona L, Garavito MF, Lozano GL, Vasquez JJ, Myers K, Fry WE (2014). De novo pyrimidine biosynthesis in the oomycete plant pathogen *Phytophthora infestans*. Gene.

[17] Loper JE, Hassan KA, Mavrodi DV, Davis EW, Lim CK, Shaffer BT (2012). Comparative genomics of plant-associated *Pseudomonas* spp.: insights into diversity and inheritance of traits involved in multitrophic interactions. PLoS Genet.

[18] Mavrodi DV, Mavrodi OV, Parejko JA, Bonsall RF, Kwak YS, Paulitz TC (2012). Accumulation of the antibiotic phenazine-1-carboxylic acid in the rhizosphere of dryland cereals. Appl Environ Microbiol.

[19] Morohoshi T, Wang WZ, Suto T, Saito Y, Ito S, Someya N, Ikeda T (2013). Phenazine antibiotic production and antifungal activity are regulated by multiple quorum-sensing systems in *Pseudomonas chlororaphis* subsp. *aurantiaca* StFRB508. J Biosci Bioeng.

[20] Xu S, Pan X, Luo J, Wu J, Zhou Z, Liang X, He Y, Zhou M (2015). Effects of phenazine-1-carboxylic acid on the biology of the plant-pathogenic bacterium *Xanthomonas oryzae* pv. *oryzae*. Pestic Biochem Phys.

[21] Thomashow LS, Weller DM (1988). Role of a phenazine antibiotic from *Pseudomonas fluorescens* in biological control of *Gaeumannomyces graminis* var. *tritici*. J Bacteriol.

[22] Puopolo G, Masi M, Raio A, Andolfi A, Zoina A, Cimmino A, Evidente A (2013). Insights on the susceptibility of plant pathogenic fungi to phenazine-1-carboxylic acid and its chemical derivatives. Nat Prod Res.

[23] Gurusiddaiah S, Weller D, Sarkar A, Cook R (1986). Characterization of an antibiotic produced by a strain of *Pseudomonas fluorescens* inhibitory to *Gaeumannomyces graminis* var. *tritici* and *Pythium* spp. Antimicrob Agents Ch.

[24] Morrison CK, Arseneault T, Novinscak A, Filion M (2016). Phenazine-1-carboxylic acid production by *Pseudomonas fluorescens* LBUM636 alters *Phytophthora infestans* growth and late blight development. Phytopathology.

[25] Mavrodi DV, Blankenfeldt W, Thomashow LS. Phenazine coumpounds in fluorescent *Pseudomonas* spp. biosynthesis and regulation. Annu Rev Phytopathol. 2006:417–45. 10.1146/annurev.phyto.44.013106.145710 16719720

[26] Arseneault T, Goyer C, Filion M (2013). Phenazine production by *Pseudomonas* sp. LBUM223 contributes to the biological control of potato common scab. Phytopathology.

[27] St-Onge R, Gadkar VJ, Arseneault T, Goyer C, Filion M (2011). The ability of *Pseudomonas* sp. LBUM 223 to produce phenazine-1-carboxylic acid affects the growth of *Streptomyces scabies*, the expression of thaxtomin biosynthesis genes and the biological control potential against common scab of potato. FEMS Microbiol Ecol.

[28] Arseneault T, Filion M (2017). Biocontrol through antibiosis: exploring the role played by subinhibitory concentrations of antibiotics in soil and their impact on plant pathogens. Can J Plant Pathol.

[29] Arseneault T, Goyer C, Filion M (2015). *Pseudomonas fluorescens* LBUM223 increases potato yield and reduces common scab symptoms in the field. Phytopathology.

[30] Götz S, García-Gómez JM, Terol J, Williams TD, Nagaraj SH, Nueda MJ (2008). High-throughput functional annotation and data mining with the Blast2GO suite. Nucleic Acids Res.

[31] Dennis G, Sherman BT, Hosack DA, Yang J, Gao W, Lane HC, Lempicki RA (2003). DAVID: database for annotation, visualization, and integrated discovery. Genome Biol.

[32] Abraham A, Philip S, Jacob MK, Narayanan SP, Jacob CK, Kochupurackal J (2015). Phenazine-1-carboxylic acid mediated anti-oomycete activity of the endophytic *Alcaligenes* sp. EIL-2 against *Phytophthora meadii*. Microbiol Res.

[33] Huang H, Sun L, Bi K, Zhong G, Hu M (2016). The effect of phenazine-1-carboxylic acid on the morphological, physiological, and molecular characteristics of *Phellinus noxius*. Molecules.

[34] Jacob C, Jamier V, Ba LA (2011). Redox active secondary metabolites. Curr Opin Chem Biol.

[35] Roy S, Poidevin L, Jiang T, Judelson HS (2013). Novel core promoter elements in the oomycete pathogen *Phytophthora infestans* and their influence on expression detected by genome-wide analysis. BMC Genomics.

[36] Andersson ME, Nordlund P (1999). A revised model of the active site of alternative oxidase. FEBS Lett.

[37] Abrahamian M, Ah-Fong AM, Davis C, Andreeva K, Judelson HS (2016). Gene expression and silencing studies in *Phytophthora infestans* reveal infection-specific nutrient transporters and a role for the nitrate reductase pathway in plant pathogenesis. PLoS Pathog.

[38] Ammar GA, Tryono R, Döll K, Karlovsky P, Deising HB, Wirsel SG (2013). Identification of ABC transporter genes of *Fusarium graminearum* with roles in azole tolerance and/or virulence. PLoS One.

[39] Amaro TM, Thilliez GJ, Motion GB, Huitema E (2017). A perspective on CRN proteins in the genomics age: evolution, classification, delivery and function revisited. Front Plant Sci.

[40] Resjö S, Ali A, Meijer HJ, Seidl MF, Snel B, Sandin M (2014). Quantitative label-free phosphoproteomics of six different life stages of the late blight pathogen *Phytophthora infestans* reveals abundant phosphorylation of members of the CRN effector family. J Proteome Res.

[41] Judelson HS, Ah-Fong AM (2010). The kinome of *Phytophthora infestans* reveals oomycete-specific innovations and links to other taxonomic groups. BMC Genomics.

[42] Jones JDG, Dangl JL (2006). The plant immune system. Nature.

[43] Sharma P, Jha AB, Dubey RS, Pessarakli M. Reactive oxygen species, oxidative damage, and antioxidative defense mechanism in plants under stressful conditions. Journal of botany. 2012;2012

[44] Küpper FC, Gaquerel E, Boneberg E-M, Morath S, Salaün J-P, Potin P (2006). Early events in the perception of lipopolysaccharides in the brown alga *Laminaria digitata* include an oxidative burst and activation of fatty acid oxidation cascades. J Exp Bot.

[45] Matsuzawa A, Saegusa K, Noguchi T, Sadamitsu C, Nishitoh H, Nagai S (2005). ROS-dependent activation of the TRAF6-ASK1-p38 pathway is selectively required for TLR4-mediated innate immunity. Nat Immunol.

[46] Matsuzawa A (2017). Thioredoxin and redox signaling: roles of the thioredoxin system in control of cell fate. Arch Biochem Biophys.

[47] Maruyama T, Araki T, Kawarazaki Y, Naguro I, Heynen S, Aza-Blanc P (2014). Roquin-2 promotes ubiquitin-mediated degradation of ASK1 to regulate stress responses. Sci Signal.

[48] Roquigny R, Arseneault T, Gadkar VJ, Novinscak A, Joly DL, Filion M (2015). Complete genome sequence of biocontrol strain *Pseudomonas fluorescens* LBUM223. Genome Announc.

[49] Forbes GA (1997). Manual for laboratory work on *Phytophthora infestans*, CIP's Training Manual EDN Quito.

[50] Daayf F, Adam L, Fernando WGD (2003). Comparative screening of bacteria for biological control of potato late blight (strain US-8), using in-vitro, detached-leaves, and whole-plant testing systems. Can J Plant Pathol.

[51] Mortazavi A, Williams BA, McCue K, Schaeffer L, Wold B (2008). Mapping and quantifying mammalian transcriptomes by RNA-Seq. Nat Methods.

[52] Robinson MD, McCarthy DJ, Smyth GK (2010). edgeR: a bioconductor package for differential expression analysis of digital gene expression data. Bioinformatics.

[53] Dillies M-A, Rau A, Aubert J, Hennequet-Antier C, Jeanmougin M, Servant N (2013). A comprehensive evaluation of normalization methods for Illumina high-throughput RNA sequencing data analysis. Brief Bioinform.

[54] Huang DW, Sherman BT, Lempicki RA (2008). Systematic and integrative analysis of large gene lists using DAVID bioinformatics resources. Nature protoc.

[55] Huang DW, Sherman BT, Lempicki RA (2008). Bioinformatics enrichment tools: paths toward the comprehensive functional analysis of large gene lists. Nucleic Acids Res.

[56] Saeed A, Sharov V, White J, Li J, Liang W, Bhagabat N (2003). TM4: a free, open-source system for microarray data management and analysis. BioTechniques.

[57] Saeed A, Bhagabati NK, Braisted JC, Liang W, Sharov V, Howe EA (2006). TM4 microarray software suite. Method Enzymol.

